# Influence of Seminal Metals on Assisted Reproduction Outcome

**DOI:** 10.1007/s12011-022-03256-w

**Published:** 2022-05-11

**Authors:** Rubí Rodríguez-Díaz, Raquel Blanes-Zamora, Rebeca Vaca- Sánchez, Jorge Gómez-Rodríguez, Arturo Hardisson, Dailos González-Weller, Ángel J. Gutiérrez, Soraya Paz, Carmen Rubio, E González-Dávila

**Affiliations:** 1 Human Reproduction Unit, Canary Islands University Hospital, San Cristobal de La Laguna, Spain; 2 Obstetrics and Gynecology, University of La Laguna, San Cristobal de La Laguna, Tenerife, Spain; 3 Toxicology, University of La Laguna, San Cristobal de La Laguna, Tenerife, Spain; 4 Mathematics, Statistics and Operations Research, University of La Laguna, San Cristobal de La Laguna, Tenerife, Spain

**Keywords:** Metal, Semen analysis, Obesity, ICSI, Embryo quality, Pregnancy

## Abstract

Increased levels of metal ions in human seminal fluid have a significant correlation with male fertility. Few publications explain the effect of metals in semen and their influence on assisted reproductive treatments. Semen parameters and the levels of twenty-two metals were measured in the seminal fluid of 102 men attended in a Reproductive Unit. Metals were determined by optical emission spectrophotometry. A statistical relationship was found between spermiogram and iron, which was lower than expected in pathological spermiograms (*p* = 0.032); zinc (*p* = 0.066), calcium (*p* = 0.047), and magnesium (*p* = 0.048) mean levels were higher in normozoospermics. More days of sexual abstinence correlates with higher seminal zinc (*p* = 0.001) and magnesium levels (*p* = 0.002). Lower vanadium values were found to be associated with higher fertilization rates (*p* = 0.039). Higher values of lead (*p* = 0.052) and vanadium (*p* = 0.032) were obtained in patients who did not reach 100% embryo cleavage rate. Aluminium (*p* = 0.042) and sodium (*p* = 0.002) were found in lower amounts associated with better blastocyst rates. The implantation rate shows an inverse association with women’s age and iron and calcium content, compared to magnesium and sodium which presented a significant direct association with this percentage. A significant direct relationship was found between the positive evolution of pregnancy and the values of zinc (*p* = 0.004), calcium (*p* = 0.013), potassium (*p* = 0.002), and magnesium (*p* = 0.009). The study confirms that zinc, iron, calcium, sodium, aluminium, magnesium, vanadium, and lead have positive–negative effects on reproduction and support the analysis of metals in semen as a new line of study on male fertility with implications for reproductive outcomes.

## Introduction

Many factors affect semen production such as stress, nutrition, trauma, obesity, tobacco smoking, alcohol consumption, or exposure to toxics, such as polychlorinated biphenyls, saturated fats, and metals [1]. Toxins and pollutants cause infertility by impairing endocrine function or spermatogenesis, via the production of reactive oxygen species (ROS) [2]. It is known that ROS are required in low amounts for a normal sperm function, but, in excess, they can affect sperm motility, morphology, and viability [3]. In order to protect sperm cells from oxidative stress, seminal fluid (SF) contains enzymatic antioxidants, particularly, superoxide dismutase (SOD), glutathione peroxidase (GPx), and catalase (CAT), as well as non-enzymatic antioxidants such as calcium (Ca), iron (Fe), zinc (Zn), and selenium (Se) [4].

When studying metals in semen, it is essential to consider that some metals are necessary, others are toxic, and some are toxic in excess but needed in smaller quantities [5]. The levels of different metals in semen may have a significant correlation with male fertility [6], and it is of interest to know the benefits and adverse effects of each metal on spermatogenesis, motility, fertilization capability, and embryo development [7], as well as on the implantation rate. In this regard, sperm function greatly depends on the ion exchange between cells and the environment in which they are immersed. These mechanisms are controlled by several channels, exchangers, and active transport systems acting on the plasma membrane of cells. These transport systems play a decisive role in triggering events that are essential for sperm fertilization, including capacitation, hyperactivation, and acrosomal reaction, among others. Some of these ion transporters only exist in sperm and are structural variants or isoforms, different to those present in somatic cells. These processes involve the participation of sodium (Na), potassium (K), and Ca ions [8] which highlights the importance of Na, K, and Ca in sperm fertility potential and has opened up a line of research on the subject when targeting male contraception [9].

Other relevant metals are magnesium (Mg), Fe, and Zn. Mg has been proved to be essential for sperm capacitation, acrosome reaction, and hyperactive motility in spermatozoa [10]. Fe acts as an antioxidant by being a cofactor of enzyme catalase, but, when elevated, it is associated with sperm damage and contributes to an increased lipid peroxidation in sperm plasma membranes, as well as affecting motility [5]. Zn acts as an antioxidant agent and increases sperm membrane fluidity, potentiating spermatozoa fertilization capacity [11], but, at toxic levels, it can have a negative effect on sperm quality [12].

On the other hand, there are metals that can be toxic for reproduction, as is the case of aluminium (Al) [13]. Aghashahi et al. [14] showed that Al increased the level of lipid peroxidation in spermatozoa and reduced its total antioxidant capacity. These results support the possibility that the harmful effects of Al on the vital sperm parameters are induced through oxidative stress (OS).

Although there are currently few publications that explain the action of metals in semen and their influence on assisted reproductive treatments (ART), several studies have shown that chronic exposure to metals can affect embryo development in ART [15].

The present study examines the association between the presence of twenty-two metals in semen and sperm quality, as well as its influence on ART results in terms of fertilization rate, embryo quality, implantation, and pregnancy rates.

## Material and Methods

### Samples

A prospective study was conducted with 102 consecutive males attended for initial evaluation in the Human Reproduction Unit of the Hospital Universitario de Canarias, in Tenerife, between February and April 2018, who underwent a semen analysis and metal detection and, subsequently, an IVF/ICSI treatment in 2019 and 2020 (*n* = 92) (Table 1). According to the results of the semen analysis [16], the subjects were categorized into two groups: forty-one participants with pathological spermiograms (40.2%), while the remaining sixty-one (59.8%) had a normal semen analysis and constituted the control group.

**Table 1:** Characteristics of males at the time of semen analysis

	Spermiogram		*p*-value	Total (*N* = 102)
	Normal (*N* = 61)	Pathological (*N* = 41)		
Age (years)	38.0 ± 5.4	38.0 ± 6.2	0.994	38.0 ± 5.7
Abstinence (days)*	3 (1.25; 4)	2 (1.5; 4)	0.569	3 (1.5; 4)
Abstinence, *n* (%)			0.890	
Few	15 (25)	10 (24)		25 (25)
Adequate	42 (70)	28 (68)		70 (69)
High	3 (5)	3 (7)		6 (6)
Weight (kg)	80.1 ± 11.0	83.1 ± 16.8	0.284	81.3 ± 13.6
Height (m)	1.77 ± 0.07	1.76 ± 0.08	0.638	1.76 ± 0.08
BMI (kg/m^2^)	25.8 ± 3.5	27.3 ± 5.2	0.094	26.4 ± 4.3
BMI, *n* (%)			0.025	
Low/normal	23 (38)	13 (32)		36 (36)
Overweight	35 (58)	19 (48)		54 (54)
Obese	2 (3)	8 (20)		10 (10)
Smoker, *n* (%)	25 (41)	9 (22)	0.074	34 (33)
Drinking, *n* (%)	40 (66)	27 (66)	0.977	67 (66)
Drug abuse, *n* (%)	1 (2)	1 (2)	0.775	2 (2)
Metal exposure, *n* (%)			0.376	
No	24 (40)	22 (54)		46 (45)
Intermediate	17 (28)	8 (20)		25 (25)
High	19 (32)	11 (27)		30 (30)
Infertility, *n* (%)			< 0.001	
Unknown	28 (46)	5 (12)		33 (33)
Female	23 (38)	2 (5)		25 (24)
Male	2 (3)	23 (56)		25 (24)
Mix	7 (11)	11 (27)		18 (18)
Preservation	1 (2)	-		1 (1)
Residence, *n* (%)			0.102	
Metropolitan	30 (49)	12 (29)		42 (41)
Outskirts	22 (36)	18 (44)		40 (39)
Minor islands	9 (15)	11 (27)		20 (20)

Data show mean ± standard deviation or frequency (percentage) except * median (P_25_; P_75_)

*BMI* body mass index

### Treatment of the Samples

Sample selection and clinical protocols were as previously described [17]. The study of seminal parameters was performed according to the WHO guidelines [16], and the following metals were measured in seminal fluid (SF): Al, boron (B), barium (Ba), Ca, cadmium (Cd), cobalt (Co), chrome (Cr), copper (Cu), Fe, K, lithium (Li), Mg, manganese (Mn), molybdenum (Mo), Na, nickel (Ni), lead (Pb), silica (Si), tin (Sn), strontium (Sr), vanadium (V), and Zn. Measurement was performed on each SF, using inductively coupled plasma optical emission spectrometry (ICP-OES) [17]. The limits of detection and quantification for each level of seminal metal are shown in Table 2.

**Table 2:** Limits of detection (LOD) and quantification (LOQ) for each level of seminal metal

Metal/wavelength	LOD mg/l	LOQ mg/l
Al (167.0 nm)	0.0040	0.012
B (249.7 nm)	0.0030	0.012
Ba (455.4 nm)	0.0010	0.005
Ca (317.9 nm)	0.5800	1.955
Cd (226,5 nm)	0.0003	0.001
Co (228.6 nm)	0.0006	0.002
Cr (267.7 nm)	0.0030	0.008
Cu (327.3 nm)	0.0040	0.012
Fe (259.9 nm)	0.0030	0.009
K (769.9 nm)	0.5650	1.884
Li (670.8 nm)	0.0050	0.013
Mg (279.1 nm)	0.5830	1.943
Mn (257.6 nm)	0.0020	0.008
Mo (202.0 nm)	0.0007	0.003
Na (589.6 nm)	1.0970	3.655
Ni (231.6 nm)	0.0007	0.003
Pb (220.0 nm)	0.0003	0.001
Si (185.0 nm)	0.0020	0.006
Sn (189.9 nm)	0.0110	0.027
Sr (407.7 nm)	0.0007	0.003
V (310.2 nm)	0.0010	0.005
Zn (206.2 nm)	0.0020	0.007

The body mass index (BMI) was 26.4 ± 4.3 kg/m^2^, 54% of the men were overweight, and 10% were obese. Smoking was present in 33% of the men, and 66% reported they occasionally drank alcohol.

Female patients underwent a controlled ovarian stimulation (COS) to retrieve the oocytes. The antagonist protocol was used: pharmacological stimulation with recombinant gonadotropins began on day 2 of the cycle, with the administration of a variable dose of 225–300 IU of rFSH (Puregon®, Organon, France or Gonal-F®, Merck Serono, France) associated or not with 100–150 IU of urinary gonadotropin HMG (Menopur®). Once the main follicle reached 14 mm in diameter, the GnRh antagonist was added subcutaneously, on a daily basis, starting with 0.25 mg of ganirelix or cetrorelix (Ganirelix, Orgalutran ®, Organon, France; Cetrorelix, Cetrotide ®, Serono, France). When the follicles were at least 17 mm, final maturation was carried out with rHCG (Ovitrelle ® 250 µg solution for injection in a pre-filled pen, choriogonadotropin alfa, Merck Serono, Bari, Italy).

The ovum pick up (OPU) was performed 36 h after the administration of rHCG by transvaginal puncture, under ultrasound vision. Once the oocytes had been retrieved, a semen sample was collected on the day of the OPU and subsequently processed for the IVF procedure. The oocytes were then inseminated, either by IVF or ICSI technique according to indication, prior to fertilization and subsequent embryo division taking place.

Fertilization rate (FR) was assessed at 18 h post insemination and was defined as the percentage of fertilized oocytes with respect to the number of mature oocytes, provided at least one had matured (MII). Embryo cleavage rate (CR) was established as the number of embryos divided embryos with respect to fertilized embryos, provided that at least one had been fertilized. A good prognosis for CR was considered to be 100% of the embryo cohort. Good embryo quality (GEQ) was established according to the ASEBIR (Association for the Study of the Biology of Reproduction) parameters for ART cycles. Embryo quality grades A and B on day 3 of division were considered good quality embryos [18]. A cycle is considered of good prognosis when the value of GEQ is greater than 66.7% of the total embryo cohort.

The achieved blastocyst rate (BR) was also considered a measure of the cycle’s degree of quality. Over the total number cycles, sixty-one had at least one cultured embryo by days 5–6, and the cycle was considered of good prognosis when at least 50% of the cultured embryos reached the blastocyst stage.

The embryo implantation rate (IR) was defined as the percentage of embryos transferred to the uterus resulting in a pregnancy. Pregnancy rate (PR) was defined as the number of cycles with at least one gestational sac among the number of cycles with embryo transfer (ET); 106 fresh ET were performed in a total of fifty-five patients and sixty-six frozen embryo transfers (FET) in a total of thirty-six patients.

### Data Analysis

Data were summarized as relative frequencies for categorical variables, means ± standard deviation for normally distributed variables, and medians (interquartile range IQR, P_25_; P_75_) for non-normal data. Comparisons were performed using Pearson’s chi-square test, Kruskal–Wallis test, or Mann–Whitney *U* test and ANOVA or Student’s *t* according to the type of variable and number of groups to be compared. The degree of relationship between continuous variables was calculated using the Pearson or Spearman correlation coefficient according to their distributions.

The cutoff point or threshold of metal content to discriminate between the successes of the different reproductive phases was established by applying receiver operating characteristics (ROC) curves and choosing the point according to the Youden index criterion. Subsequently, logistic regression with Wald’s backward variable selection method was used to model success in the different phases including variables from the spermiogram, as well as metal content. ROC curves and the area under the curve (AUROC) were included.

A generalized linear mixed model with binary logistic distribution and logit link, including random intercept and slope, was applied in the study of embryo implantation to consider couples with different numbers of interventions. The selection of variables was carried out using a backward type procedure (*p*-out = 0.10) to prevent a possible muti-collinearity effect. The value of Akaike information criterion (AIC_c_) and percentage of concordance was provided.

SPSS V 25 (IBM SPSS Statistics) and MedCalc V 19.5 (MedCalc Software Ltd.) were used. A value of *p* ≤ 0.05 was considered significant.

## Results

### SF and Metals

In our study group, 59.8% of the semen samples were normozoospermic and 40.2% were pathologic. All semen samples were found to contain Ca, K, Na, and Zn, while Ba, Co, Mn, and Mo were not detected, and the remaining metals were only detected in some patients (Table 3). A high correlation was obtained between some metals, such as Al and Ni (*r*_*s*_ = 0.558; *p* < 0.001) and between Ca and K (*r*_*s*_ = 0.833; *p* < 0.001). In others, this relationship was weak, as was the case between Cu and Fe (*r*_*s*_ = 0.248; *p* = 0.012) or Sr and Fe (*r*_*s*_ =  − 0.245; *p* = 0.013), while in many others, no relationship was observed, for example, between Sr and Al (*r*_*s*_ =  − 0.028; *p* = 0.777) or Fe and Zn (*r*_*s*_ = 0.009; *p* = 0.931).

**Table 3:** Metal contents in the spermiogram

	Spermiogram		*p*-value	Total (*N* = 102)
	Normal (*N* = 61)	Pathological (*N* = 41)		
Essentials, *n* (%)				
Co	-	-		-
Mo	-	-		-
Cr	1 (2)	1 (2)	0.775	2 (2)
Cu	58 (95)	38 (93)	0.682	95 (94)
Fe	61 (100)	38 (93)	0.032	99 (97)
Zn	61 (100)	41 (100)		102 (100)
Mn	-	-		-
Cu (mg/kg)	0.59 ± 0.34	0.54 ± 0.36	0.521	0.57 ± 0.34
Fe (mg/kg)	0.61 ± 0.31	0.66 ± 0.64	0.618	0.63 ± 0.47
Zn (mg/kg)	96.2 (65.3)	75.3 (37.0)	0.066	87.8 ± 56.4
Cu (mg/kg)*	0.52 (0.34; 0.79)	0.48 (0.34; 0.64)	0.434	0.50 (0.34; 0.74)
Fe (mg/kg)*	0.55 (0.36; 0.84)	0.42 (0.30; 0.74)	0.321	0.51 (0.33; 0.82)
Zn (mg/kg)*	86.7 (46.1; 116.3)	71.6 (46.4; 100.0)	0.201	82.0 (46.5; 111.3)
Fe, *n* (%)			0.383	
25% lower (< 0.332)	12 (20)	13 (32)		25 (25)
50% central	33 (54)	19 (46)		52 (50)
25% higher (> 0.824)	16 (26)	9 (22)		25 (25)
Zn, *n* (%)			0.314	
25% lower (< 46.46)	15 (25)	10 (24)		25 (25)
50% central	28 (46)	24 (59)		52 (50)
25% higher (> 111.26)	18 (29)	7 (17)		25 (25)
No essentials, *n* (%)				
Ba	-	-	-	-
Al	44 (72)	31 (76)	0.872	75 (74)
B	8 (13)	8 (20)	0.553	16 (16)
Cd	1 (2)	1 (2)	0.775	2 (2)
Li	1 (2)	-	0.598	1 (1)
Ni	10 (16)	6 (15)	0.811	16 (16)
Pb	26 (43)	20 (49)	0.682	46 (45)
Sr	35 (57)	23 (56)	0.898	58 (57)
V	49 (80)	32 (78)	0.977	81 (79)
Al (mg/kg)	2.45 ± 4.20	2.91 ± 4.24	0.589	2.64 ± 4.20
B (mg/kg)	0.08 ± 0.25	0.11 ± 0.31	0.580	0.10 ± 0.28
Ni (mg/kg)	0.04 ± 0.19	0.03 ± 0.07	0.587	0.04 ± 0.15
Pb (mg/kg)	0.02 ± 0.03	0.02 ± 0.03	0.720	0.02 ± 0.03
Sr (mg/kg)	0.07 ± 0.09	0.07 ± 0.10	0.824	0.07 ± 0.09
V (mg/kg)	0.44 ± 0.38	0.47 ± 0.38	0.714	0.46 ± 0.37
Al (mg/kg)*	0.38 (0; 1.04)	0.32 (0.11; 8.84)	0.877	0.34 (0; 7.43)
B (mg/kg)*	0 (0; 0)	0 (0; 0)	0.419	0 (0; 0)
Ni (mg/kg)*	0 (0; 0)	0 (0; 0)	0.842	0 (0; 0)
Pb (mg/kg)*	0 (0; 0.04)	0 (0; 0.03)	0.779	0 (0; 0.04)
Sr (mg/kg)*	0.06 (0; 0.10)	0.06 (0; 0.09)	0.909	0.06 (0; 0.09)
V (mg/kg)*	0.41 (0.13; 0.64)	0.50 (0.18; 0.77)	0.624	0.42 (0.15; 0.69)
Macroelements, *n* (%)				
Ca	61 (100)	41 (100)		102 (100)
K	61 (100)	41 (100)		102 (100)
Mg	51 (84)	34 (83)	0.928	85 (83)
Na	61 (100)	41 (100)		102 (100)
Ca (mg/kg)	259.0 ± 102.1	227.2 ± 56.9	0.073	246.2 ± 87.8
K (mg/kg)	844.7 ± 274.4	773.7 ± 161.8	0.139	816.1 ± 237.3
Mg (mg/kg)	73.8 ± 52.1	60.0 ± 32.5	0.137	68.2 ± 45.6
Na (mg/kg)	2134.7 ± 256.6	2161.8 ± 241.2	0.593	2145.6 ± 249.6
Ca (mg/kg)*	238.7 (183.2; 311.8)	231.6 (181; 279.2)	0.253	236.4 (182.9; 289.4)
K (mg/kg)*	763.3 (664.1; 997.1)	779.6 (635.0; 895.4)	0.399	770.6 (647.6; 936.6)
Mg (mg/kg)*	75.1 (43.7; 95.4)	67.7 (47.9; 85.5)	0.222	69.1 (46.3; 89.3)
Na (mg/kg)*	2092 (1969; 2315)	2135 (1990; 2341)	0.597	2133 (1980; 2328)
Ca, *n* (%)			0.011	
25% lower (< 182.9)	15 (25)	10 (24)		25 (25)
50% central	25 (41)	27 (66)		52 (50)
25% higher (> 289.4)	21 (34)	4 (10)		25 (25)

Data show mean ± standard deviation or frequency (percentage) except * median (P_25_; P_75_)

A statistical relationship was observed when analysing metals such as Fe, Zn, Ca, and Mg and seminal parameters. In addition, all men with normal sperm analyses presented Fe in semen, compared to men diagnosed with altered spermiograms, where this percentage was 92.7% (*χ*^2^_1_ = 4.59; *p* = 0.032). In these forty-one samples, no Fe was detected in three men and twenty-one had less than < 0.50 mg/kg Fe. No samples were identified between 0.50 and 0.61 mg/kg Fe, and seventeen had more than 0.61 mg/kg. The level of Fe in spermiogram in the first quartile (25% lower), measuring 0.33 mg/kg, was found in more pathological samples than expected (*χ*^2^_2_ = 6.921; *p* = 0.031), as men with pathological sperm concentrations have higher levels of Fe (90.6% vs 97%) (*χ*^2^_1_ = 6.48; *p* = 0.011).

Regarding Zn, the mean level of this metal was higher in men with normal spermiograms (96.22 ± 65.29 mg/kg) compared to pathological ones (75.29 ± 37.01 mg/kg) (*t*_100_ = 1.86; *p* = 0.066) without reaching statistical significance. Furthermore, men with normal sperm concentrations tended to have higher Zn levels (95.42 ± 62.39 vs 71.18 ± 35.82 mg/kg) (*t*_100_ = 2.04; *p* = 0.044) and reached statistical significance.

In addition, a significant relationship was identified between spermiogram and seminal Ca levels (*t*_100_ = 2.01; *p* = 0.047), which were higher in normozoospermic males (259.0 ± 102.1 mg/kg) versus pathologic spermiograms (227.2 ± 56.9 mg/kg). The same applies to sperm concentration and Ca (*t*_100_ = 2.50; *p* = 0.014), which was higher in normal sperm concentrations (258.1 ± 97.3 mg/kg) versus oligozoospermia (220.2 ± 55.2 mg/kg). Moreover, sperm concentration and Mg (*t*_100_ = 2.00; *p* = 0.048) showed a statistical relationship. In addition, the mean average Mg in semen was higher in normal sperm counts (73.5 ± 49.1 mg/kg) versus oligozoospermia (56.6 ± 34.4 mg/kg). Higher levels of Zn, Ca, and Mg were formed in normozoospermia, while Fe was higher in pathologic spermiogram.

On the other hand, when analysing the influence of obesity on semen quality, it was observed that 3.3% of normozoospermic men were obese, while the percentage reached 19.5% (*χ*^2^_1_ = 7.307; *p* = 0.013) in the pathologic semen group. A statistical relationship with Fe was found when considering the association between BMI and the presence of metals. Thus, in obese males (BMI ≥ 30.0 kg/m^2^), the percentage of men with Fe in SF (80%) was lower than expected (97%) (*χ*^2^_2_ = 11.302; *p* = 0.001). In addition, a greater number of days of sexual abstinence showed a correlation with higher levels of seminal n (*r*_*s*_ = 0.375; *p* = 0.001) and Mg (*r*_*s*_ = 0.311; *p* = 0.002).

As for the influence of toxic habits on the content of metals in SF, no significant relationship was observed between the presence/absence of metals and smoking or alcohol consumption. Conversely, residential and occupational exposures were found to have an effect on Ca and Al. Therefore, a significant difference in Ca levels (*F*_4;92_ = 2.742; *p* = 0.033) was observed among males living on the outskirts of metropolitan areas and being occupationally exposed. More specifically, men with some levels of occupational exposure had higher concentrations of Ca in SF than those with no occupational exposure. Furthermore, Al values were found to be positively related to metal exposure (*F*_2;96_ = 4.190; *p* = 0.018).

### Assisted Reproductive Treatment

The study was carried out with ninety-two males; ninety-six started ART, but one did not attend the day of OPU, and the oocytes did not mature in three females. Forty-seven participants (51.1%) had a normal spermiogram, while the other forty-five (48.9%) were pathologic. Furthermore, fifty-eight males (63.0%) had normal sperm concentrations versus thirty-four (37.0%) provided pathological samples.

### Oocytes

The number of oocytes per OPU ranged between one and twenty-seven, with the median being six oocytes. On the other hand, the number of oocytes obtained was inversely related to the woman’s age (*r* =  − 0.288; *p* = 0.004) and a high positive correlation was found between the number of MII oocytes and fertilization rate (*r* = 0.938; *p* < 0.001).

### Fertilization Rate

Median fertilization rate (FR) was 74.3% (55.5; 100). Twenty-seven participants (29%) presented 100% FR and only seven patients (7.6%) had an FR lower than 33.3%, with three patients (3.2%) having an FR of 0%. The mean average difference between MII and fertilized oocytes is 1.87 ± 2.08 oocytes, varying between 0 and 12.

**Fig. 1:**
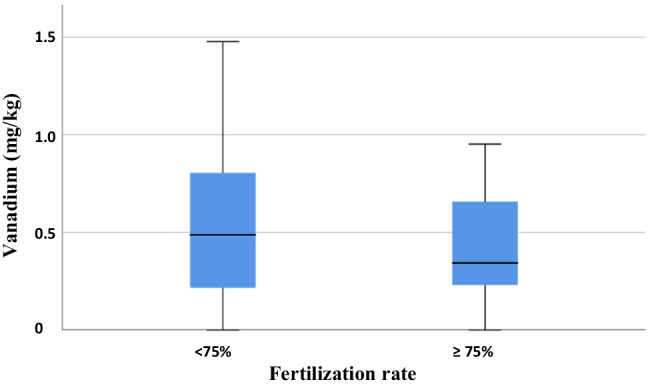
Vanadium content versus fertilization rate. When V was lower than 0.7 mg/kg, an increase in FR was observed, with an OR of 3.06 (CI_95%_ 1.05, 8.94). The final model was ln (*p*/(1 − *p*)) =  − 3.520 + 2.583 (if technique = ICSI) + 1.118 (if V < 0.7 mg/kg) with *p* being equal to the probability of an FR above 75% and ln logarithmic function

A significant relationship was found between fertilization and V, and a trend was observed with Fe. The effect of V is noteworthy among all the studied metals. The group with an FR above 75% presented lower V values compared to those with rates below 75% (*p* = 0.039). In this latter group, 33.3% presented V values higher than 0.7 mg/kg compared to 15.8% who presented V it in the group with an FR greater than 75% (OR = 2.67 CI_95%_ 1.01, 7.52; *p* = 0.048). When V was lower than 0.7 mg/kg, an increase in the FR was observed, with an OR of 3.06 (CI_95%_ 1.05; 8.94). When ICSI was the technique used, an increased rate was also observed compared to IVF with an OR 13.24 (CI_95%_ 1.62; 107.87). The ROC curve showed an AUROC of 0.604 (CI_95%_ 0.505; 0.719) (Fig. 1). The probability of having an FR higher than 75% was 54.5% when ICSI was performed and V < 0.7 mg/kg. In other cases, this probability would decline to 50% or less. The final model was ln (*p*/(1 − *p*)) =  − 3.520 + 2.583 (if technique = ICSI) + 1.118 (if V < 0.7 mg/kg) with *p* equal to a probability of an FR above 75% and ln the logarithmic function.

Although not significant, the trend high Fe values showed towards high FR is worth mentioning, 31.6% of the samples with an FR above 75% exceeding 0.68 mg/kg compared to only 20.4% in the group with a rate below 75% (*p* = 0.164) (OR 1.80 CI_95%_ 0.70; 4.67). The ROC curve shows an AUROC of 0.593 (CI_95%_ 0.477; 0.710).

### Cleavage Rate

Three of the participants did not present MII oocytes, and the total number of interventions studied was eighty-nine. Seventy-eight participants (87.6%) reached a CR of 100%, and only six (6.7%) presented a rate equal to or lower than 80% (Table 4).

**Table 4:** Characteristics and metal contents according to embryo cleavage rate

	Cleavage rate		*p*-value	Total (*N* = 89)
	< 100% (*N* = 11)	= 100% (*N* = 78)		
Female				
Age (years)	33.6 ± 4.63	35.0 ± 4.00	0.284	34.8 ± 4.08
BMI (kg/m^2^)	22.9 ± 2.78	23.9 ± 3.58	0.425	23.8 ± 3.50
Male				
Age (years)	37.3 ± 7.39	38.0 ± 5.28	0.675	37.9 ± 5.54
BMI (kg/m^2^)	25.5 ± 2.96	25.6 ± 3.26	0.965	25.6 ± 3.21
Spermiogram			0.118	
Normal	8 (72.7)	36 (46.2)		44 (49.4)
Pathological	3 (27.3)	42 (53.8)		45 (50.6)
Count			0.194	
Normal	9 (81.8)	46 (59.0)		55 (61.8)
Pathological	2 (18.2)	32 (41.0)		34 (38.2)
Technique			0.032	
IVF	4 (36.4)	7 (9.0)		11 (12.4)
ICSI	7 (63.6)	68 (87.2)		75 (84.3)
Others	-	3 (3.8)		3 (3.4)
Metals				
Essentials, *n* (%)				
Co	-	-		-
Mo	-	-		-
Cr	-	2 (2.6)	0.767	2 (2.2)
Cu	10 (90.9)	73 (93.6)	0.558	83 (93.3)
Fe	11 (100)	76 (97.4)	0.767	87 (97.8)
Zn	11 (100)	78 (100)		89 (100)
Mn	-	-		-
Cu (mg/kg)*	0.81 (0.43; 0.94)	0.52 (0.34; 0.74)	0.105	0.53 (0.34; 0.81)
Fe (mg/kg)*	0.45 (0.30; 0.69)	0.43 (0.33; 0.72)	0.808	0.43 (0.33; 0.70)
Zn (mg/kg)*	75.2 (43.9; 99.2)	75.4 (42.3; 97.3)	0.704	75.2 (42.7; 98.3)
No essentials, *n* (%)				
Ba	-	-		-
Al	7 (63.6)	59 (75.6)	0.465	66 (74.2)
B	1 (9.1)	11 (14.1)	0.649	12 (13.5)
Cd	1 (9.1)	2 (2.6)	0.330	3 (3.4)
Li	-	-		-
Ni	-	7 (9.0)	0.590	7 (7.9)
Pb	7 (63.6)	30 (38.5)	0.105	37 (41.6)
Sr	6 (54.5)	36 (46.2)	0.750	42 (47.2)
V	10 (90.9)	62 (79.5)	0.683	72 (80.9)
Al (mg/kg)*	0.42 (0; 0.64)	0.32 (0.18; 0.71)	0.935	0.33 (0; 0.68)
B (mg/kg)*	0 (0; 0)	0 (0; 0)	0.585	0 (0; 0)
Ni (mg/kg)*	0 (0; 0)	0 (0; 0)	0.304	0 (0; 0)
Pb (mg/kg)*	0.04 (0; 0.06)	0 (0; 0.03)	0.052	0 (0; 0.04)
Sr (mg/kg)*	0.12 (0; 0.29)	0 (0; 0.09)	0.145	0 (0; 0.09)
V (mg/kg)*	0.68 (0.47; 0.95)	0.41 (0.21; 0.69)	0.032	0.44 (0.21; 0.70)
Macroelements, *n* (%)				
Mg	9 (81.8)	64 (82.1)	0.985	73 (82.0)
Ca (mg/kg)*	238.2 (164.2; 282.1)	230.2 (178.4; 285.5)	0.751	231.6 (176.2; 283.3)
K (mg/kg)*	809.9 (628.4; 1094.9)	760.7 (636.4; 944.8)	0.995	761.5 (636.0; 947.3)
Mg (mg/kg)*	69.3 (41.7; 87.7)	63.7 (42.3; 87.2)	0.861	65.5 (42.3; 87.2)
Na (mg/kg)*	2071.0 (1909.1; 2262.6)	2135.2 (1987.0; 2329.6)	0.210	2135.2 (1972.7; 2324.5)

Data show mean ± standard deviation or frequency (percentage) except * median (P_25_; P_75_)

*BMI* body mass index

A statistical relationship was found between CR and Pb and V. Those who did not reach 100% CR presented higher Pb values (*p* = 0.052). Of these, 63.6% presented Pb compared to 38.5% in the group reaching 100% division. This same relationship was observed for V, with higher values in the group that did not reach 100% (*p* = 0.032), and V being present in 90.9% of the samples compared to 79.5% in the group reaching 100%.

**Fig. 2:**
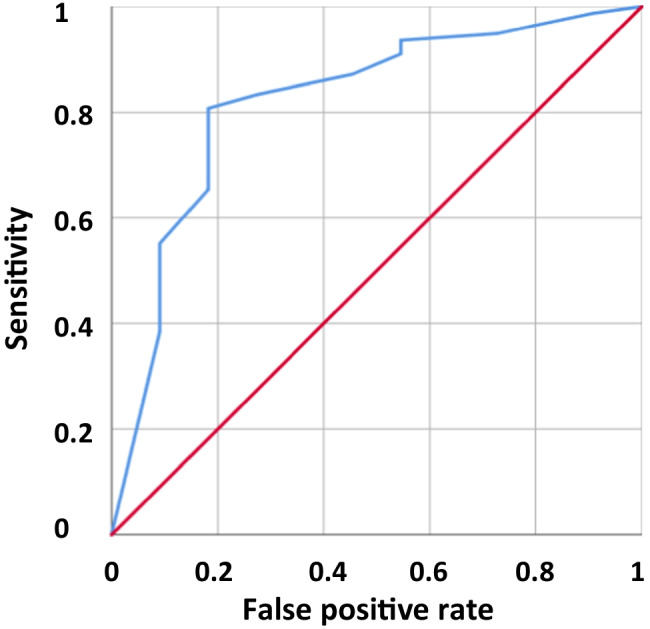
ROC curve for the probability of the cleavage rate equal to 100%. The final model was ln (*p*/(1 − *p*)) =  − 2.625 + 1.703 (if technique = ICSI) + 1.906 (if V < 0.7 mg/kg) + 1.890 (if Cu < 0.8 mg/kg) + 1.423 (if Pb < 0.041 mg/kg) with *p* being equal to probability of CR reaching 100%. This model give an AUROC of 0.821 (CI_95%_ 0.678; 0.963; *p* = 0.001)

The final model was ln (*p*/(1-*p*)) =  − 2.625 + 1.703 (if technique = ICSI) + 1.906 (if V < 0.7 mg/kg) + 1.890 (if Cu < 0.8 mg/kg) + 1.423 (if Pb < 0.041 mg/kg) with *p* equal to the probability of CR reaching 100%. This model gives an AUROC of 0.821 (CI_95%_ 0.678; 0.963; *p* = 0.001) (Fig. 2).

### Embryo Quality

GEQ results were obtained in the eighty-nine participants who had at least one MII oocyte, ranging from 0 to 9 embryos, with the median being 2 (0; 4). GEQ was not reached in eleven participants (12.4%), a single GEQ was reached in twenty-six participants (29.2%), and five or more GEQ were achieved in twelve participants (13.5%). The rate exceeded 50% in half of the cases and reached a rate of 66.7% in 25% of the cases, although 25% had a GEQ rate lower than 33.3%. A 100% rate was reached in twelve participants.

The only factors that seemed to be come close to significance were Al, and the result of the spermiogram. As for Al, 84.2% of the samples with a GEQ rate lower than 50%, presented this metal (*p* = 0.087), falling to 66.7% in those having the best rates (*p* = 0.054). The cutoff point for Al was 0.58 mg/kg, indicating there is an OR of 3.826 (CI_95%_ 1.015; 14.415) having a GEQ rate higher than 66.7% when presenting Al < 0.58 mg/kg compared to those with Al ≥ 0.58 mg/kg.

Similarly, the probability of presenting a high GEQ rate increased when the spermiogram was normal. In the group presenting rates lower than 66.7%, the percentage of pathological spermiogram was 56.9% compared to 33.3% in the group with rates higher than 66.7% (*p* = 0.105). The AUROC was 0.717 (CI_95%_ 0.605; 0.828; *p* = 0.002); this model includes Al < 0.58 mg/kg.

### Blastocyst Rate

BR had a median of 33.3% (0; 50). In other words, 50% of the cultures had blastocyst formation rates lower than 33.3% and 25% had a BR greater than 50%.

As for metals, Al and Na are noteworthy. Both metals were found in lower amounts with a better BR. Thus, in the group presenting BR < 50%, Al was < 0.288 mg/kg in 26.3% of the cycles compared to the group with a better BR where this percentage reached 52.2% (*p* = 0.042). Regarding Na, the group with the lowest BR below 50% presented Na < 2242 mg/kg in 42.1% of the cycles, compared to 82.9% in the group with the highest BR above 50% (*p* = 0.002). Once again, the results were better when the ICSI technique was performed.

### Embryo Transfer

A total of 106 ETs were performed in fifty-five participants, forty-two (39.6%) from fresh cycle and sixty-four (60.4%) from frozen cycle. ET cycles (55.7%) received one embryo and 44.3% received two embryos. More transfers of two embryos were recorded in fresh cycles compared to frozen cycles (57.1% vs. 35.9%; *p* = 0.032).

The mean number of fresh ETs per couple was 1.93 (SD 1.10). The figures did not differ significantly between couples who achieved implantation and those who did not. Sixty-four FETs were performed in thirty-six participants. A single ET was performed in eighteen participants, with a median of two transferred embryos.

### Implantation

Implantation was achieved in twenty-eight (50.9%) couples compared to twenty-seven (49.1%) where it failed. No significant differences (*p* = 0.629) were observed between fresh and FET cycles in terms of embryo implantation, seventy-seven (72.6%) with zero implanted, twenty-four (22.6%) with one implanted, and five (4.7%) with two implanted (Table 5).

**Table 5:** Characteristic and metal contents in the semen depending on whether the transferred embryos achieve implantation or not

	Implantation		***p***-value	Total (***N*** = 55)
	No **(*N*** = 27)	Yes (***N*** = 28)		
Female				
Age (years)	35.7 ± 3.17	32.6 ± 4.40	0.005	34.1 ± 4.10
BMI (kg/m^2^)	23.7 ± 3.17	23.8 ± 3.94	0.930	23.8 ± 3.55
Male				
Age (years)	39.5 ± 5.30	36.9 ± 5.67	0.078	38.2 ± 5.61
BMI (kg/m^2^)	25.3 ± 2.75	26.4 ± 3.77	0.229	25.8 ± 3.29
Spermiogram			0.898	
Normal	13 (48.1)	13 (46.4)		26 (47.3)
Pathologic	14 (51.9)	15 (53.6)		29 (52.7)
Sperm count			0.785	
Normal	17 (63.0)	16 (57.1)		33 (60.0)
Pathologic	10 (37.0)	12 (42.9)		22 (40.0)
No. of embryo transfer	1.93 ± 1.07	1.93 ± 1.15	0.993	1.93 ± 1.10
Embryo transfer, *n* (%)			0.580	
1	13 (48)	13 (47)		26 (47)
2	6 (22)	9 (32)		15 (27)
3	5 (19)	2 (7)		7 (13)
4 or more	3 (11)	3 (14)		7 (13)
Metals				
Essential, *n* (%)				
Co	-	-		-
Mo	-	-		-
Cr	2 (7.4)	-	0.236	2 (3.6)
Cu	26 (96.3)	25 (89.3)	0.611	51 (92.7)
Fe	27 (100)	26 (92.9)	0.491	53 (96.4)
Zn	27 (100)	28 (100)		55 (100)
Mn	-	-		-
Cu (mg/kg)*	0.57 (0.34; 0.83)	0.45 (0.36; 0.59)	0.178	0.53 (0.35; 0.74)
Fe (mg/kg)*	0.63 (0.38; 0.88)	0.36 (0.31; 0.57)	0.007	0.45 (0.33; 0.68)
Zn (mg/kg)*	85.8 (40.1; 115.5)	65.2 (44.3; 98.7)	0.775	80.2 (42.4; 110.6)
No essential, *n* (%)				
Ba	-	-		-
Al	22 (81.5)	19 (67.9)	0.355	41 (74.5)
B	3 (11.1)	7 (25.0)	0.295	10 (18.2)
Cd	2 (7.4)	-	0.236	2 (3.6)
Li	-	-		-
Ni	3 (11.1)	1 (3.6)	0.352	4 (7.3)
Pb	13 (48.1)	14 (50.0)	0.891	27 (49.1)
Sr	14 (51.9)	16 (57.1)	0.789	30 (54.5)
V	20 (74.1)	22 (78.6)	0.758	42 (76.4)
Al (mg/kg)*	0.46 (0.26; 8.79)	0.30 (0; 0.41)	0.024	0.34 (0; 0.62)
B (mg/kg)*	0 (0; 0)	0 (0; 0.24)	0.211	0 (0; 0)
Ni (mg/kg)*	0 (0; 0)	0 (0; 0)	0.262	0 (0; 0)
Pb (mg/kg)*	0 (0; 0.04)	0.01 (0; 0.04)	0.914	0 (0; 0.04)
Sr (mg/kg)*	0.06 (0; 0.09)	0.07 (0; 0.15)	0.358	0.06 (0; 0.11)
V (mg/kg)*	0.41 (0; 0.68)	0.45 (0.19; 0.78)	0.446	0.43 (0.16; 0.74)
Macroelement, *n* (%)				
Mg	23 (85.2)	22 (78.6)	0.729	45 (81.8)
Ca (mg/kg)*	234.9 (179.1; 293.9)	230.8 (177.9; 283.6)	0.749	234.9 (179.1; 289.9)
K (mg/kg)*	777.6 (635.4; 942.3)	768.0 (640.8; 946.1)	0.801	776.0 (636.7; 942.3)
Mg (mg/kg)*	69.9 (43.8; 94.3)	63.4 (42.4; 85.5)	0.398	69.4 (43.8; 88.8)
Na (mg/kg)*	2080.8 (1965.0; 2258.7)	2263.4 (2097.1; 2350.6)	0.027	2199.5 (1989.2; 2329.5)

Data show mean ± standard deviation or frequency (percentage) except * median (P_25_; P_75_)

*BMI* body mass index

It was observed that, when two embryos were transferred, implantation was achieved in 29.8% of the participants compared to 25.4% when only one embryo was transferred. Most significantly, within the ET, when two embryos were transferred, 33.3% implantation was successful compared to 22.2% when only a single embryo was transferred (*p* = 0.206).

Table 6 shows a model of the statistical relationship between implantation and Mg, Ca, Fe, and Na. This model presents a value of AIC = 868.52 with a precision of 72.6% (81.8% in non-implantation and 48.3% in implantation). Therefore, Mg (*p* = 0.017) and Ca (*p* = 0.033) showed a positive relationship, while Fe (*p* = 0.043) showed a negative relationship (less quantity, better implantation). When analysing the influence of Fe on implantation, it was observed that, in the cases where implantation failed, median Fe was 0.63 mg/kg compared to 0.36 mg/kg where implantation occurred (*p* = 0.007) (Table 6).

**Table 6:** Generalized mixed linear model for the implantation variable (binary logistics with logit link)

		s.e.	*p*-value	Odds ratio (OR)	CI_95%_ (OR)
Intercept	7.796	6.197	0.211		
Age Female	-0.236	0.115	0.042	0.790	(0.630; 0.992)
Fe	-2.137	1.041	0.043	0.118	(0.015; 0.935)
Ca	-0.034	0.016	0.033	0.967	(0.937; 0.997)
Mg	0.062	0.026	0.017	1.064	(1.011; 1.120)
Na	0.014	0.003	<0.001	1.014	(1.007; 1.021)

CI: confidence interval; s.e.: standard error.

### Pregnancy

Of the 106 ETs performed, twenty-nine resulted in pregnancies, twelve of these (41.4%) from fresh cycles, and seventeen (58.5%) from FETs. After analysing pregnancies according to the type of ET cycle used, it was observed that twelve pregnancies occurred in the forty-two (28.6%) fresh ETs, and seventeen in the sixty-four (26.6%) FETs (*p* = 0.827). Out of the twenty-nine pregnancies, 22 (75.8%) showed a positive evolution (one was a twin pregnancy) compared to seven (24.2%) pregnancy losses and one ectopic pregnancy. Thus, in the fresh cycle, eleven (91.7%) pregnancies evolved positively (one twin) compared to FETs where eleven pregnancies were also obtained, but representing 64.7% (*p* = 0.187), as five implantations (29.5%) ended in pregnancy loss and one (5.8%) in ectopic pregnancy.

A statistical significance was detected between pregnancy and Zn, Ca, K, Mg, and B, highlighting the noteworthy differences detected regarding Zn (*p* = 0.004), Ca (*p* = 0.013), K (*p* = 0.002), and Mg (*p* = 0.009), with higher values when pregnancies evolved positively. Although with a non-significant *p*-value, it should be noted that B (*p* = 0.147) was present in 31.8% of the samples that evolved positively compared to none in those that did not. Sr (*p* = 0.192) was observed in 63.6% of the samples that evolved positively while it was present in only 28.6% of the ones that did not, and Mg (*p* = 0.131) was present in 86.4% of the samples that had a positive outcome while it was found in only 57.1% of those with a negative outcome.

## Discussion

SF analysis is a simple, non-invasive procedure providing information for an accurate diagnosis. It is the body fluid of choice to detect the exact levels of metals, unlike blood, which may not be reflect the true exposure to these elements regarding the male reproductive system [19].

Human SF contains several trace elements that play important roles in normal sperm function [20]. Various metals such as Ba, Co, Mn, and Mo were not detected in any semen sample of the cohort. This does not mean they are not present, as they could be below the detection limit of the device (ICP-OES), which does not rule them out as being potentially harmful. Strong or weak correlations between some metals, such as Al and Ni, Ca and K, or Cu and Fe and Sr and Fe were also obtained. Other authors did not find the same correlations leading them to believe that such a trend may point to a common source of origin for these metals [7].

One of the main findings of this study lies in the fact significantly higher levels of Fe in pathological spermiograms were found, while levels of Zn, Ca, and Mg were detected at higher levels in normozoospermia.

As a cofactor of CAT, Fe could act as an antioxidant protector when present in physiological levels. However, Fe can also act as a prooxidant catalysing the formation of hydroxyl radicals that can attack sperm plasma membranes [21].

As already described for Fe, at a concentration of five parts per million, Fe could induce lipid peroxidation, leading to an inhibition of sperm motility, and a correlation was found between Fe and sperm damage [5].

In the study conducted by Habib et al. [22], Fe and Cd levels were significantly higher in sperm homogenate and seminal plasma of asthenozoospermic and azoospermic groups compared to their corresponding controls, suggesting that Fe and Cd might have a strong toxic effect on spermatogenesis by producing excessive oxidants and inducing apoptosis, a process that leads to male infertility by depletion of sperm concentration. A negative association between seminal Pb or Cd concentrations and sperm concentrations, sperm motility, and abnormal spermatozoa was found [23]. In the same vein, different authors reported that SF Cd and Pb were significantly higher in azoospermic and oligospermic men when compared to normozoospermic men, and these authors suggested that environmental exposure to both metals may contribute to the development of poor sperm quality and infertility in men [24].

Accordingly, all normozoospermic men in this study presented Fe in SF, although Fe was found in higher levels than expected in pathological spermiograms. In addition, other authors [5, 7] found increased levels of Fe in asthenozoospermic males. Regarding pathological semen in the present study, in the first quartile, when Fe levels where less than 25%, the semen sample showed a greater probability of being pathological than expected indicating that the main role of Fe takes places when its values are between a minimum and a maximum. Thus, Fe values below or above these limits could be harmful to sperm function, hence the importance of keeping a balance [7, 21].

The above also draws attention to the fact that high Fe levels in SF correlates with a high FR, even when it is not statistically significant, and a negative correlation was observed between Fe and IR results. Thus, 31.6% of samples with an FR above 75% exceed 0.68 mg/kg, compared to only 20.4% in the group with an FR below 75%. No publication in the literature search was found relating Fe to FR.

Zn is another important metal, which is essential for male reproductive potential, and is necessary for normal fertilization [11], above all in the pre-fertilization process, from sperm capacitation, zygote activation, and the final zona pellucida reaction [25], to the post-fertilization stages [21]. It has been well documented that a deficiency in Zn is associated with an increase in apoptosis and, therefore, a reduction in sperm count, given that Zn is a cofactor of SOD that is one of the most important enzymes for antioxidant defence [11] enhancing the formation of spermatozoa and cell motility [26]. However, publications about the role of Zn in male fertility report different findings and conclusions. Zhao et al. [27] concluded that Zn levels in the seminal plasma of infertile males were significantly lower than that of normal males, and thus, Zn deficiency may cause sperm dysfunction and male infertility, but, on the other hand, a highly toxic content of Zn could have a negative effect on sperm quality [12, 28, 29]. In the present study, agreeing with other authors [5, 7, 28], Zn was present in all semen samples, since a significant relationship was discovered between higher Zn levels and better seminal quality.

Regarding the remaining metals, no influence was observed on FR and there are no current studies on the subject. It is well known that Zn plays a role in the initial stages of germ cell development and spermatogenesis, as well as in sperm maturation, capacitation, and fertilization. Zn also acts as an antioxidant agent, besides increasing sperm membrane fluidity and potentiating spermatozoa fertilization capacity [27]. Higher levels of Zn were also observed in the present study in males with normal spermiograms with higher sperm counts and a trend to improve FR, in agreement with Kumar et al. [30], who observed slightly higher Zn levels in the SF of IVF with a positive pregnancy outcome and suggested that Zn might play a positive role in ET as well as IVF outcome. Zn plays an important part in the penetration of the sperm into the oocyte to form a mature zygote, as well as in the post-fertilization period, showing a positive correlation with pregnancy outcomes for ART, when Zn supplementation is included as part of the treatment [11].

Furthermore, an upward trend in Zn levels was observed in cases with more days of sexual abstinence which could be related to the increase in the time of absorption and storage of metals in the SF. However, the authors could not find another publication on the subject in the literature search.

Ca is another important element which acts as an intracellular second messenger and could be a mediator of oxidative damage, inducing lipid peroxidation [31]. Ca presence proved essential for sperm capacitation, acrosomal reaction, and hyperactive sperm motility [32]. Consequently, the presents study found significant differences in semen quality, which improved with higher levels of Ca. However, other authors have not reported any correlation between seminal Ca and sperm quality [20].

When studying the reproductive results in terms of FR, CR, EQ, and BR, no significant effects on these parameters were observed with decreased levels of Ca. At the time of fertilization, an increase in intracellular Ca induces sperm movement across the oocyte prompting its activation. During spermatozoa-egg interaction, Ca is injected into the oocyte triggering a signal transduction pathway and initiating activation [10, 33]. Correia et al. [34] showed that a large amount of Ca is concentrated in the head of mature spermatozoa facilitating Ca injection into the oocyte. It is possible that the levels affecting reproductive results were lower than those achieved in the cohort of the present study. Likewise, it should be noted that the best results in the study here were achieved with the ICSI technique, where the fertilization process is bypassed technically and Ca intervention is not a determinant, which could explain why no influence of Ca was observed on FR.

Regarding Mg action, an abnormal presence of Mg may affect spermatogenesis, both in sperm production and maturation and motility. As is known, ATP Mg^2+^-dependent ATPase generates the energy for sperm motility or fertilization capacity [35]; thus, decreased levels of Mg in semen have been linked to infertility [36]. In agreement with this, the asthenozoospermic samples in the present had a lower concentration of Mg.

Mg is provided by the prostate gland, and an Mg decrease in semen could be caused by an unhealthy state of the prostate gland [11, 37]. Therefore, a significant increase of Mg concentration was obtained in males with a larger period of sexual abstinence, indicating this metal is accumulated in the prostate gland. This fact has not been corroborated by other authors who did not observe any statistical differences regarding Mg levels in SF after ten days of sexual abstinence [35].

Higher levels of Zn, Ca, and Mg are present in better PR, and these three metals are also higher in normozoospermia, which is a logical result since embryo quality is also better when spermiograms are normal. In agreement with other authors [30], the present study found that the level of Zn in male SF was higher in positive ART outcomes.

It is known that K plays a crucial role in the hyperpolarization of the sperm membrane that is essential for the flow of Ca in the processes that occur in the acrosome reaction, which explains its importance in fertilization processes [20]. Although no relation was observed here between K and FR, it may influence PR.

In addition, various researchers found a significant low concentration of K in semen from infertile male compared to fertile males and a significant decrease in the level of Na in pathological groups [25]. Despite this, other authors did not report significant correlations between Na levels in seminal plasma and sperm quality [37].

However, one of the discoveries of the present study is the finding of an association between the presence of higher Na levels in semen and better IR, thus highlighting the important contribution of this element to fertility. A positive relationship was observed between Na and motile spermatozoa.

Furthermore, Mirnamniha et al. [25] reported that human semen contains several trace elements such as Cu, Zn, Ca, Na, K, Mn, and Mg, which are necessary for normal spermatogenesis, sperm maturation, motility, and capacitation, as well as normal sperm function, and although these elements are essential for the fertilization process, increased levels of certain metals, such as Mn and Cu, can be toxic for human sperm and are negatively correlated with sperm parameters. Conversely, decreased levels of some trace elements can negatively affect human reproductive health, semen quality, sperm normal function, and, as a result, fertility potency in men [25].

Another relevant finding of the study here lies in the observation that higher levels of V proved to be detrimental to IVF outcomes. The group with FR above 75% had lower V values, while men with V values higher than 0.7 mg/kg were 2.67 times more likely to have a fertilization rate below 75%. Various studies in animals have shown that V produces toxicity in the male reproductive system through oxidative stress resulting in decreased sperm count and motility, as well as a higher concentration of abnormal sperm and, therefore, a potential reduction of FR. As mentioned above, high V levels produce oxidative stress affecting sperm functionality and increasing the number of abnormal sperm [38], which may affect not only fertilization, but also CR.

It is well known that reproductive toxicity, resulting from exposure to toxic metals, may harm the success of IVF [15, 39]. The results obtained here match those of previous studies [17], as well as a prospective study, where a negative correlation was observed between Pb levels of SF and IVF success rates [40]. The reason could be that Pb can bind to oestrogen receptors and may potentially interfere with sexual steroid hormone signalling pathways and, thus, may alter embryo development [41]. Conversely, other authors have found no significant effects with Pb on CR outcomes [19, 30]. However, some authors [19] observed a higher chance of IR associated with higher concentrations of Pb in paternal semen and blood. In addition, Zhou et al. [42] detected levels of Ni, Cu, and Mo in seminal plasma that were significantly correlated with BR and GEQ. However, this relationship was not observed in the present study. The levels of Pb and Cd in SF detected by Wijesekara et al. [43] were associated with reduced sperm viability, count, motility, and normal forms, and, besides, in a meta-analysis, Lopez-Botella et al. [6] observed that higher Pb concentrations in SF were associated with lower total motile sperm and higher concentrations of Cd in SF led to lower probabilities for implantation and pregnancy rate. A lower fecundability associated with higher male Pb concentrations has also been reported; however, no conclusion could be drawn for the relationship between paternal blood Cd, Pb levels, and IVF outcomes [30].

The present study found a relationship between Al in semen and EQ, as males in the GEQ group showed a lower presence of Al with statistical significance, which was also reflected in the possibility of reaching the blastocyst stage. Nevertheless, when GEQ was achieved, no differences were observed in the evolution of the pregnancy. However, the results obtained show that a greater presence of Al in semen exerts a detrimental effect on the fertilization process, although it does not appear to impair seminal parameters. An underlying cause for the toxic effects of aluminium chloride was found to be the oxidative stress that affects the vital sperm parameters, lipid peroxidation, and the total antioxidant capacity of spermatozoa, but this effect can be reversed by antioxidant use [14].

It is noteworthy that IR has an inverse relationship with the woman’s age; Fe and Ca contents, compared to Mg and Na, have a significant direct effect on the IR. Bassey et al. [36] reported results suggesting that seminal plasma Ca and Mg play a markedly important role in male fertility and should be considered as part of the management plan for male infertility.

The present study found that the number of oocytes obtained was inversely related to the woman’s age. This agrees with most of the studies showing that age is an important risk factor for ovarian reserve: in low ovarian reserve, the number of eggs is lower, thus reducing the chances of achieving pregnancy naturally [44].

When analysing the link between lifestyle and metals, a significant relationship was found between Fe and BMI, the percentage of obese men with Fe in semen being lower than expected. This is the only relationship observed between obesity and metals. No studies were identified in the literature search linking the increase in BMI with the presence of Fe in semen. However, evidence was found pointing to an association between obesity and insulin resistance [45].

As regards, lifestyle and alcoholic beverages, including wine, can be contaminated with metals at high levels, leading to toxic effects, especially in heavy drinkers [46] which may be associated with severe disturbances in sperm production [42]. In the case of the present study, 65.7% of the males consumed alcohol, some only at weekends and others on a daily basis. Recent studies have investigated the relationship between alcohol consumption and seminal quality, with mixed results. Some of these studies have observed a relationship between alcohol consumption and decreased seminal parameters [47], while others (including the present study) did not [48], which could be due to differences in the assessment of alcohol intake and level of exposure between subjects.

Similarly, no significant relationship between metal levels, smoking, and environmental exposure was found in this study, but place of residence had a degree of influence on Ca levels. Some authors [49] agree that sperm motility is worse in heavy smokers compared to controls. However, one of the major differences with the present study is that males were categorized as heavy smokers when more than ten cigarettes a day were consumed [17] while the former considered as heavy smokers as those who smoked more than twenty-five cigarettes a day were consumed [49]. Both results could have been similar if the levels of smoking criteria coincided.

## Limitations

The study has few limitations. The first limitation of the study is the volume of semen required for metal detection, which was 0.5 ml. This was because the semen sample was used to perform a spermiogram and sperm capacitation at the same time.

Participants were included in the study because of the infertility of one partner or the other, or both, which is why this group includes fertile men and a variable range of infertility problems, including female infertility [17]. Therefore, this supports the use of this population in an epidemiologic study where the influence of environmental contaminants in male reproduction is studied.

## Conclusion

It is now clear that heavy trace metals are associated with a pattern different of effects on various reproductive endpoints, suggesting a pathophysiological pathway unique to each element [19]. There are few studies analysing the effect of heavy metals in paternal semen on ART outcomes. Therefore, more studies are needed to understand the real impact of metals on ART results. The determination of metals in semen opens up a new field in the study of male infertility, and many cases of unknown infertility could be due to metal presence or absence as well as altered concentrations in semen, with the option of performing treatments for these possible anomalies.

The study confirms the importance of Zn, Fe, Ca, Na, Al, Mg, V, and Pb in the positive–negative effects on reproduction and supports the analysis of metals in semen as a new field of study on male fertility with implications for reproductive outcomes.

## Data Availability

Data that support the findings of this study are available from the corresponding author upon reasonable request. **Ethical Standards** The authors affirm that all procedures contributing to this work comply with the ethical standards of the relevant national and institutional committees on human experimentation and with the Helsinki Declaration of 1975, as revised in 2008. The present study protocol was reviewed and approved by the institutional review board of Hospital Universitario de Canarias (Reg. No. 2017_84), and all eligible participants signed an informed consent form prior to inclusion.
